# Supplementary material for: Genetic and Molecular Basis of Heterogeneous NK Cell Responses against Acute Leukemia
Source: Cancers (Basel). 2020 Jul 16;12(7):1927. doi: 10.3390/cancers12071927 (PMC7409189; doi:10.3390/cancers12071927)

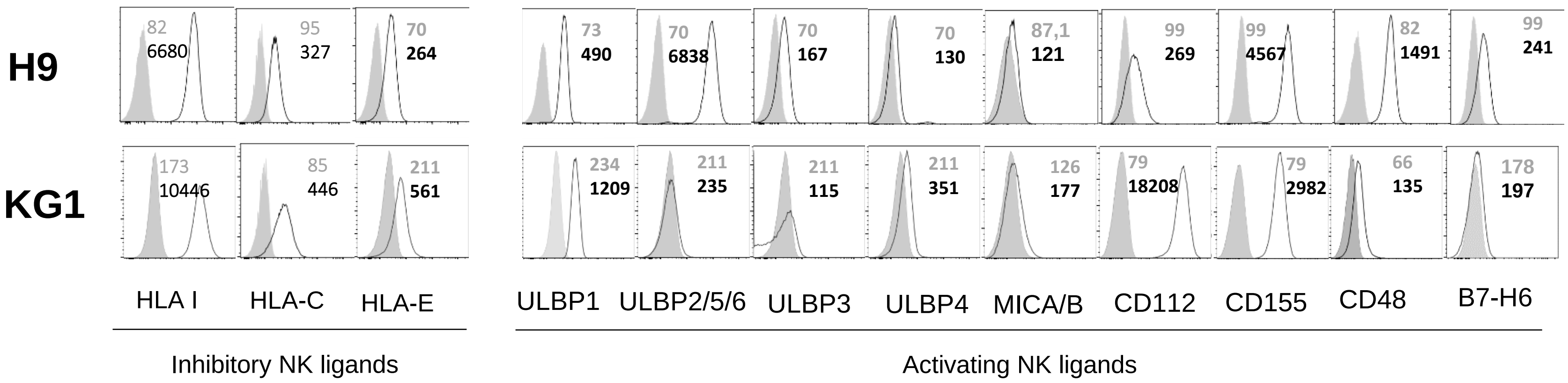

Figure S1: Immunophenotyping of H9 ALL and KG1 AML cell lines. Representative density plots illustrating inhibitory and activating NK ligand expression on H9 ALL and KG1 AML cell lines. Cell lines were stained using mAb (clear grey) and isotype control (filled grey) and were analyzed by flow cytometry. The Mean Fluorescent Intensity (MFI) of each NK ligand and the corresponding controls are indicated in the density plots.

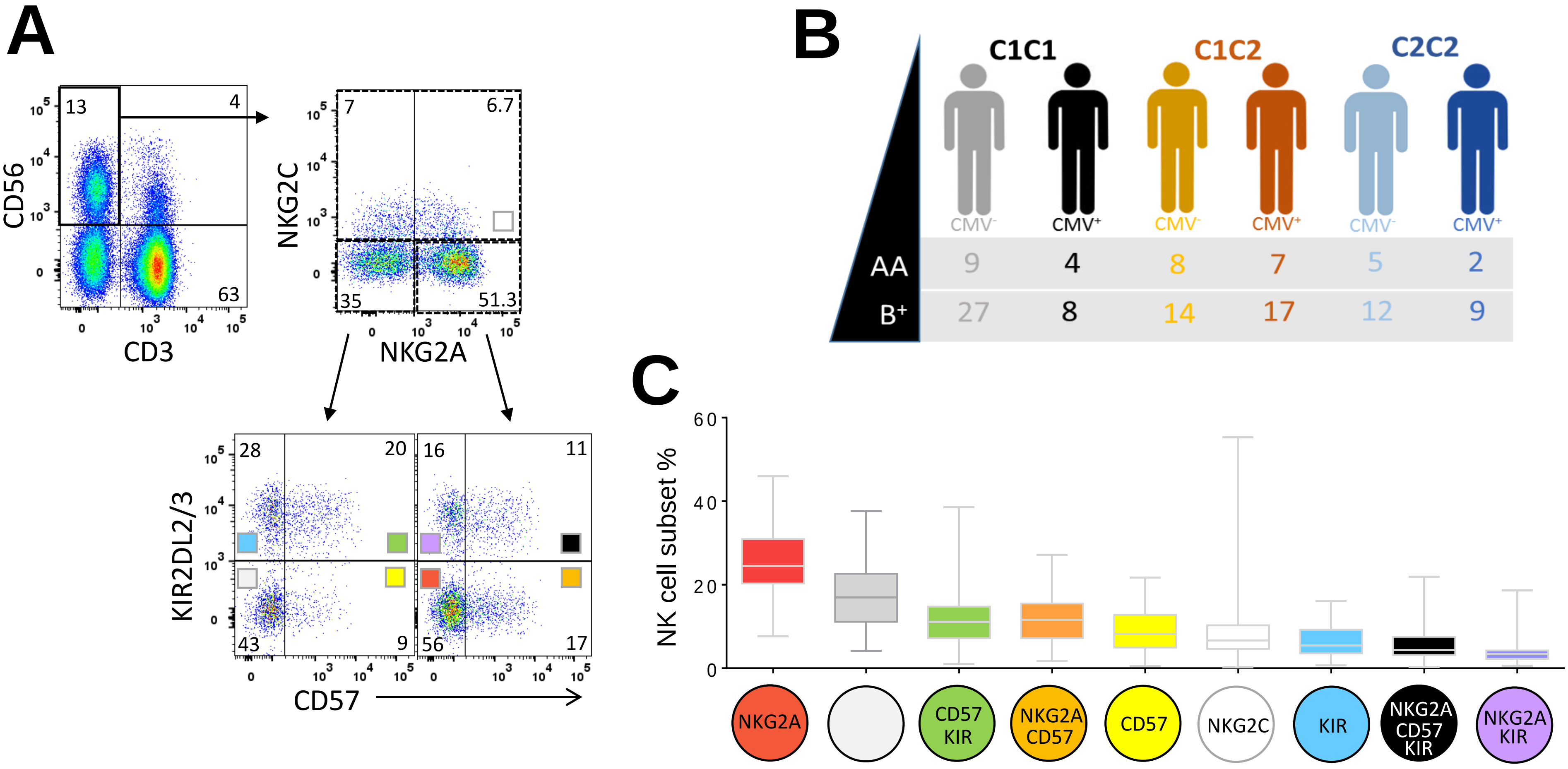

Figure S2: Flow Cytometry strategy to determine the frequency of different NK cell subsets. (A) Density plots illustrating cell targeting strategy used by multi-color flow cytometry to target 9 NK cell subsets ($CD3^-CD56^+$) based on the KIR, NKG2A, CD57 and NKG2C markers. A color code is indicated for the 9 NK cell subsets. **(B)** Distribution of blood donors (n=68) following KIR and HLA genetics and CMV status. **C)** Whisker graphs showing the frequency of the 9 NK cell subsets : $NKG2A^+KIR^-CD57^-$ (red), NKG2A+KIR+CD57- (purple), $NKG2A^+KIR^+CD57^+$ (black), $NKG2A^-KIR^+CD57^-$ (blue), $NKG2A^-KIR^+CD57^+$ (green), $NKG2A^-KIR^-CD57^+$ (yellow), $NKG2A^+KIR^-CD57^+$ (orange), $NKG2A^-KIR^-CD57^-$ (grey) and $NKG2C^+$ (white) investigated in 68 blood donors. NK cell subsets were classified from the most represented to the least represented following the same color code. Shown are mean ±SD.

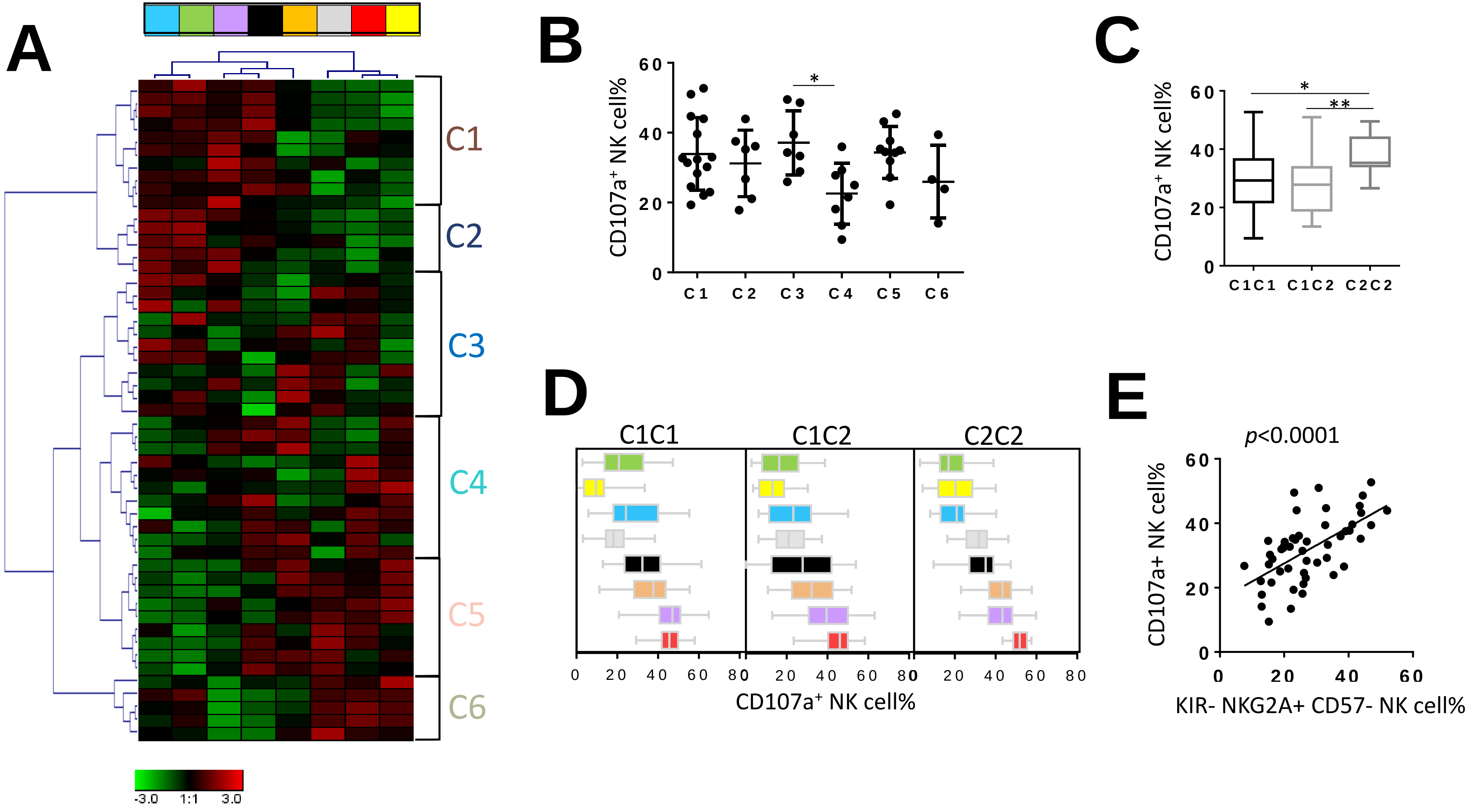

Figure S3: NKG2A^{+} NK cell subsets are the most efficient against primary ALL blasts. (A) Heatmap clustering 51 blood donors (C1 to C6) from the degranulation of 8 NK cell subsets against primary ALL COE-B blasts. Each column is dedicated to a defined NK cell subset. **(B)** Dot plots of the whole NK cell degranulation of all blood donors clustered from C1 to C6. **(C)** Whisker graphs of the whole NK cell degranulation according to the HLA-C environment (C1C1, C1C2 and C2C2) in 51 blood donors. **(D)** Whisker graphs of degranulation frequency of the 8 investigated NK cell subsets of C1C1, C1C2 and C2C2 individuals. **(E)** Correlation between frequencies of NKG2A^{+}KIR^{-}CD57^{-} NK cells and whole NK cell degranulation from 51 blood donors.

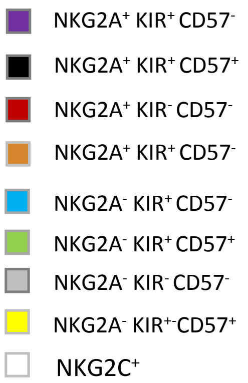

Supplement: Supplementary file 1 [file cancers-12-01927-s001.pdf]
